# Sensitivity and specificity of the ICDAS II system and bitewing radiographs for detecting occlusal caries using the Spectra™ caries detection system as the reference test in children

**DOI:** 10.1186/s12903-023-03615-6

**Published:** 2023-11-21

**Authors:** Jorge H. Caceda, Shuying Jiang, Victor Calderon, Ebingen Villavicencio-Caparo

**Affiliations:** 1 Rutgers School of Dental Medicine, Department of Pediatric Dentistry & Community Health, 110 Bergen St, Newark, NJ USA; 2 Rutgers-School of Dental Medicine, Office of Academic Affairs, 110 Bergen St, Newark, NJ USA; 3 Universidad Peruana Cayetano Heredia, School of Dentistry, Department of Medicine and Maxillofacial Surgery, Ave. Honorio Delgado 430, San Martin de Porres, Lima, Perú; 4 Universidad Católica de Cuenca Ecuador, Av. Humboldt y Av. Las Américas, Cuenca, Ecuador; 5 Universidad Peruana Cayetano Heredia, School of Dentistry, Department of Social Dentistry, Ave. Honorio Delgado 430, San Martin de Porres, Lima, Peru

**Keywords:** Sensitivity, Specificity, Caries detection, ICDAS II, Radiographs, Fluorescent camera, Children

## Abstract

**Background:**

Most studies assessing the sensitivity and specificity of caries detection methods have been performed in vitro using the histological method as the gold standard showing inconsistent values. The aim of this study was to compare the sensitivity and specificity in detecting occlusal caries using the International Caries Detection and Assessment System (ICDAS II) with the radiographic method (RM), while using the Spectra™ Caries Detection System (SCDS) as the reference test.

**Methods:**

One hundred sixty children, ages 7–12 years, participated in the study. Five zones in the occlusal surfaces of 859 primary and 632 first permanent molars were examined visually using ICDAS-II, the RM using bitewing radiographs and SCDS. The descriptive statistics of sensitivity and specificity were calculated and compared.

**Results:**

For all molars combined and for primary molars only, the sensitivity of ICDAS II was higher for detecting total caries (*p* < 0.001), caries in enamel (*p* < 0.001), and caries in dentin (*p* = 0.016), but it was not different for detecting caries in the dentin of permanent first molars (*p* = 0.214), and primary second molars (*p* = 0.761). The specificity of RM was higher for detecting total caries, caries in enamel for all molars combined and for permanent first molars (*p* < 0.001). For caries in dentin, the specificity of ICDAS II was higher for all molars combined and for primary molars only (*p* < 0.001). For total caries in primary molars only, and caries in dentin of permanent first molars only, the specificity was not different (*p* = 0.156 and *p* = 0.181 respectively).

**Conclusions:**

The sensitivity and specificity of ICDAS II and RM changes depending on whether the carious lesion compromises the enamel or dentin, and if the caries detection is performed in the primary molars or permanent first molars.

## Background

Various visual examination methods (VE) have been developed for detecting and differentiating carious lesions in the enamel or dentin [1, 2]. One of the systems used with the VE, that has been studied and compared extensively with other caries detection methods, is the International Caries Detection and Assessment System (ICDAS II) [3–8].

Another method used in the detection of dental caries is the radiographic method (RM) [9, 10]. Although bitewing radiographs are better for detecting interproximal carious lesions, they are also capable of detecting occlusal caries as demonstrated in the study by Hopcraft and Morgan, who concluded that the prevalence of interproximal and occlusal caries was underestimated when only the visual and tactile examination were used [11].

The validity and reliability of caries detection systems are based on their sensitivity and specificity in detecting a tooth with caries (true positive-sensitivity of the test) or a caries-free tooth (true negative-specificity of the test) [12, 13].

To determine the sensitivity and specificity of a diagnostic test, it is known that a gold standard test must be used. The ideal gold standard should have 100% sensitivity and 100% specificity. We do not know of any method, system or technique used in the detection of occlusal caries that has these two characteristics. Regarding the last point, it has been suggested that any caries diagnostic method used to detect caries must have sensitivity greater than or equal to 0.75 and specificity of 0.85 or greater [14].

Because it is less expensive and time consuming, most studies comparing the sensitivity, specificity, accuracy and reliability of the systems, used for detecting dental caries, have been performed in vitro, using the histological method as the reference test [15, 16]. Although the histological test gives a more controlled scenario and is a feasible option in an in vitro study, in an in vivo study, the use of the histological test requires the removal of the tooth which in many cases cannot be implemented for ethical reasons. In addition to this ethical conflict, the histological test has also been questioned as a gold standard due to the manipulation, sectioning and grinding of the tissue which might create changes in the area of the tooth that must be examined [17]. Therefore, the dilemma that an in vivo study design has in comparing the sensitivity and specificity of a caries detection method is to select a gold standard with a similar or better diagnostic ability than the histological test. Then the question is: what system can be used to compare the sensitivity and specificity of a caries detection method in an in vivo study?

Technological advances in the field of optics have fostered the development of new caries detection instruments such as the Quantitative Light-Induced Fluorescence, the DIAGNOdent™, and light fluorescent cameras such as the Spectra™ Caries Detection System (SCDS) (Air Techniques, Inc. Melville, NY, USA), which facilitates the detection of caries using high energy light on the tooth’s surface making cariogenic bacteria fluoresce red and healthy enamel fluoresce green. These systems have made it possible to detect changes in the structure of the enamel that could not be seen with VE or RM. Moreover, conventional techniques have been shown to have poor sensitivity and are only capable of detecting dental caries in the most advanced stages [18, 19].

Various studies comparing fluorescent cameras such as the SCDS, and a similar caries detection system named Vista Proof with conventional and histological tests have shown that these systems have similar or better sensitivity and specificity, suggesting that they can be used as a gold standard in an in vivo study instead of the histological test [1, 20–28].

In clinical and epidemiological studies, identifying a test with high sensitivity and specificity not only helps detect groups with a high prevalence of the disease or at risk of developing the disease, but also helps to implement strategies to prevent the disease, distribute financial resources efficiently, and improve access to the health system [29, 30].

In developed countries, the use of the light fluorescence system (LFS) has become an available method to detect, diagnose and monitor incipient carious lesions in a clinical setting [15, 31]. In these countries, the LFS may be an easy option to be implemented, whereas in underserved communities of developing countries, the implementation and use of these systems are almost impossible due to their high cost and lack of basic services such as electricity and running water.

The aims of this study were to compare the sensitivity and specificity of The International Caries Detection and Assessment System (ICDAS II) and radiographic method (RM) in detecting occlusal carious lesions while using the Spectra™ Caries Detection System (SCDS) as the reference test, and to determine if the sensitivity and specificity varies according to the type of tooth examined, and whether the carious lesion is compromising the enamel only or the dentin.

## Methods

This descriptive and comparative study was approved and performed following the regulations of the Institutional Ethics Committee of the Universidad Peruana Cayetano Heredia (UPCH) (code number: 60917). The sample size of 140 children was estimated based on the prevalence of dental caries (90%) in the population with an error and precision of 5%. To avoid a reduction in the sample size during the time of the study, an additional 15% of children for a total of 161 children ages 7–12 years from an underserved community located in Ventanilla-Callao-Peru, were included in the study. The term underserved was based on the following criteria: (1) the community does not have access to basic public services, such as running water, electricity, and a health care system, and (2) is in a peri-urban area with poor transportation access [32, 33].

An informed consent and assent including the purpose, benefits and risks involved in the study were obtained from the parents and participants in the study.

### Detection of occlusal caries

**Fig. 1:**
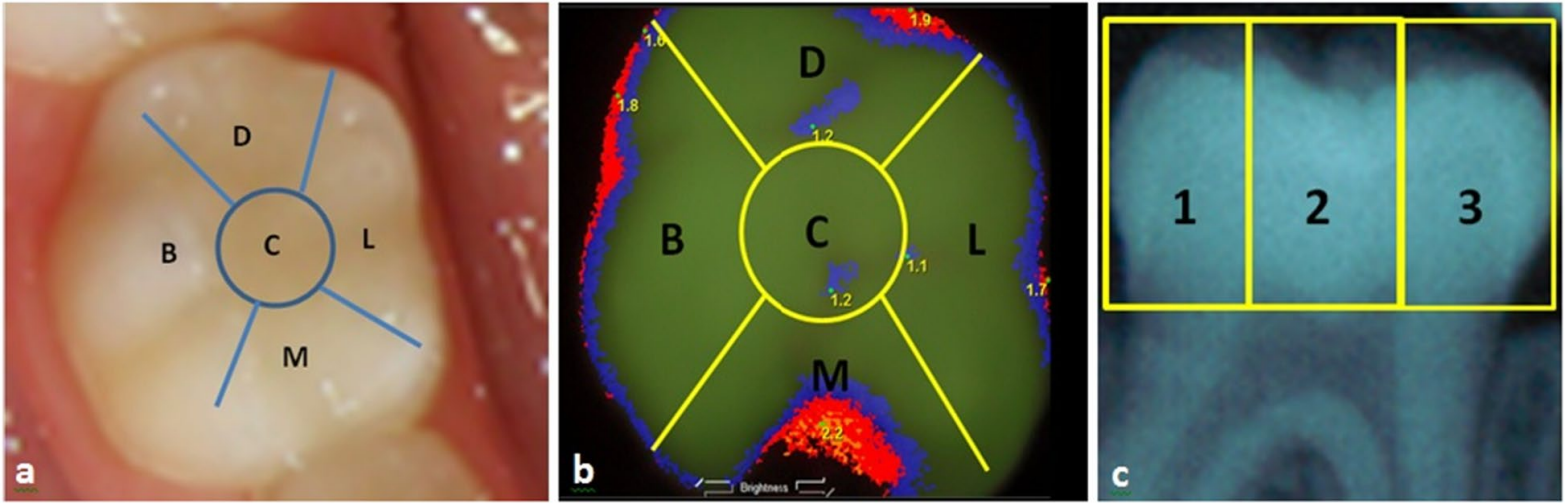
Occlusal zones evaluated in the detection of caries (**a**) ICDAS II System, (**b**) Spectra^TM^ Caries Detection System, and (**c**) Radiographic Method

All VE using the ICDAS-II criteria and SCDS were performed by one of the authors (JC). Calibration in the use of the ICDAS-II codification and criteria to detect occlusal caries was performed through the learning program sponsored by the ICDAS foundation [34]. The intra-examiner reliability at the end of the learning process was 90%. Children with any chronic medical conditions and those who could not tolerate the non-invasive VE, RM, and the SCDS examinations were excluded from the study. The detection of occlusal caries using ICDAS II and SCDS was performed on the same day. The teeth of all children were cleaned with a disposable toothbrush and water, and then dried with a piece of gauze to avoid any distortion due to the reflection, absorption or scattering of the light. To avoid a bias associated with the visualization of the image on the occlusal surface when SCDS was used, all VE were performed first. A #4 mouth mirror and a combination of natural and LED light were used to examine the occlusal surfaces of all primary first and second molars, and permanent first molars. Caries detection using ICDAS II was performed in five zones of the occlusal surface following a geometric format previously designed and following the codification and criteria established by ICDAS.org and modified by the principal investigator to fulfill the codification used in the study (Fig. 1a, b, Table 1).

**Table 1:** Criteria and scores used for data analysis using the ICDAS II, the radiographic method (RM) and the Spectra™ Caries Detection System (SCDS)

Score	**ICDAS II Criteria**^**a**^	**Score**	**RM Criteria**^**b**^	**Score**	**SCDS Criteria**^**d**^
0	Healthy tooth (no evidence of caries)	0	Healthy tooth (no radiolucency)	0 - 1.0	Healthy tooth (no evidence of caries)
1	Change in enamel (opacity or discoloration) visible in the pits and fissures after drying the tooth’s surface	1	Radiolucency in the outer half of the enamel	1.1 – 1.5	Initial caries into the enamel
2	Change in enamel (opacity or discoloration) visible before and after drying the tooth	2	Radiolucency in the inner half of the enamel	1.6 – 2.0	Caries in enamel that extend to the junction of enamel and dentin
3	Localized enamel breakdown without signs of dentin involvement	3	Radiolucency in the outer half of the dentin	2.1 – 2.5	Caries into the dentin
4	Dark discoloration shadow from dentin	4	Radiolucency in the inner half of dentin	> 2.5	Caries deep into the dentin
5	Caries with evidence of demineralization and dentin exposure	5	Radiolucency with initial pulp involvement		
6^e^	Extensive carious lesion with visible dentin that compromise more than half of the tooth surface	6^e^	Radiolucency with severe pulp involvement		
7^e^	Temporary filling	66^e^	The occlusal zone was not captured in the radiographic imagen		
77^e^	Resin based composite filling	7^e^	Restored tooth		
8^e^	Amalgam filling	777^e^	Root remnants		
88^e^	Occlusal zone cannot be evaluated	88^e^	Occlusal zone cannot be evaluated		
9^e^	Missing tooth	9^e^	There are no values, or the tooth is not present		
999^e^	There are no values to record				
**Criteria and scores used for data analysis**					
Type of Caries	VM score^a^		RM score^b^	SCDS score^c^	
No caries	0		0	0 – 1.0	
Caries in enamel	1,2,3		1,2	1.1 – 2.0	
Caries in dentin	4,5,6		3,4,5,6	> 2.0	

^a^Criteria and scores shown in the table were modified from the original data published by Ismail AI et al. [4]

^b^Criteria and scores shown in the table were modified from the original data published by Otis L and Sherman R [35].

^c^Scores modified based on SCDS criteria

^d^Criteria and scores shown in the table were modified from the original data article published by Graye et al. [26]

^e^Modified by the authors

**Fig. 2:**
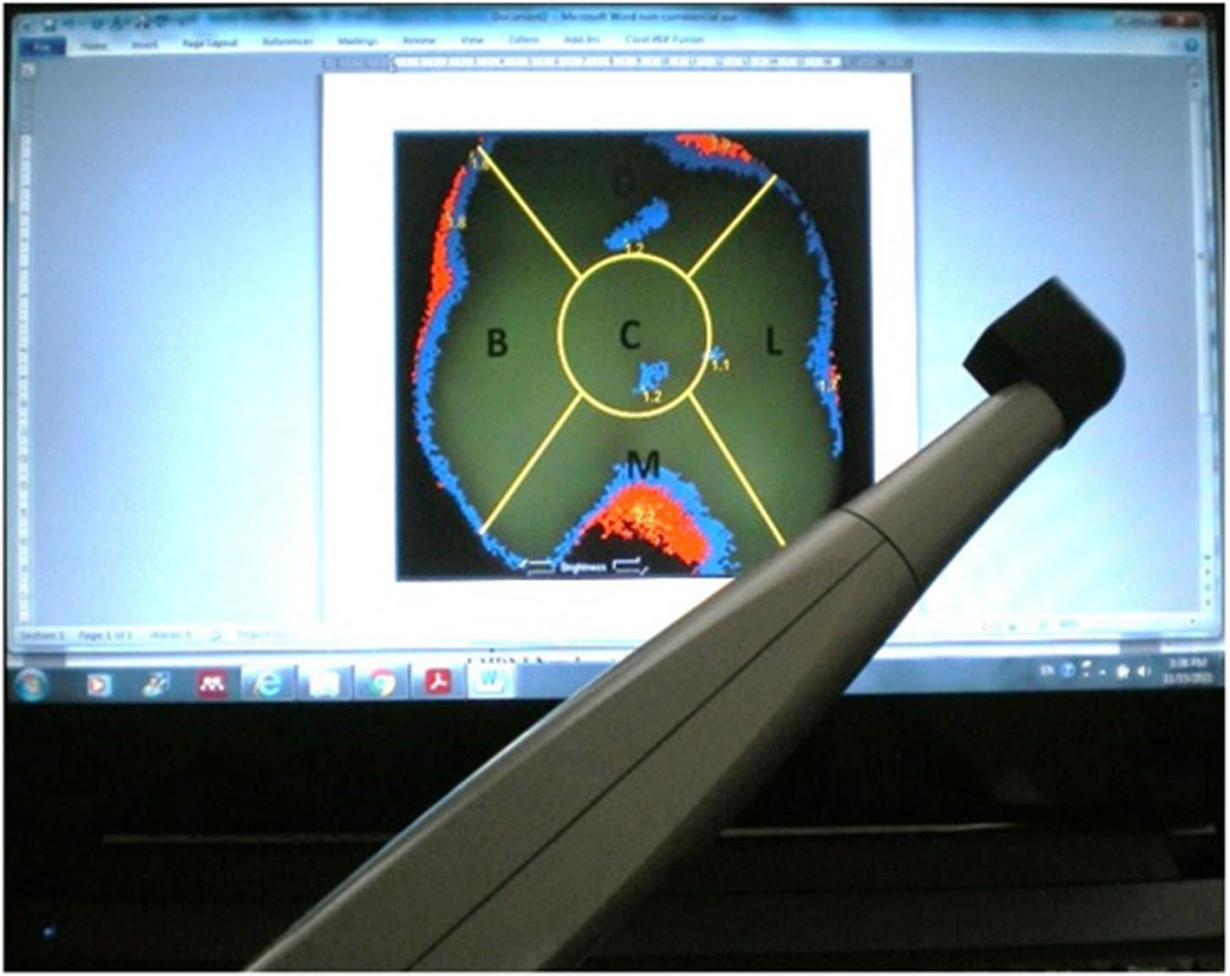
Spectra™ light fluorescent system showing the 10 mm black plastic separator

The detection of occlusal caries with SCDS was performed using a 10 mm black plastic separator (Fig. 2). This separator avoided the penetration of external light and allowed for the same distance between the lens of the camera and the occlusal surface, preventing distortions of the image. All images were transferred to the Visix® (Air Technique, Inc., Melville, NY) dental imaging processor and stored in a file generated by the program for further interpretation and analysis. The criteria established for the detection of occlusal carious lesions using SCDS was based on the manufacturer’s recommendations.

Conventional radiograph Kodak films (Ultra-speed DF-58 and DF-54) and a calibrated Kodak radiographic equipment model CS2100 attached to a mobile dental unit (kilovoltage: 60, milliamp: 7, exposure time: 0.25–0.40 s, focal point: 7 mm and a cone of 20 cm) from the Department of Community Dentistry-UPCH were used to take two bitewing radiographs in two consecutive days on all children that participated in the caries detection examination with ICDAS II and SCDS. All radiographs were taken by an experienced dental radiologist using the parallel technique with zero-degree angulation and processed manually the same day in a dark room with a safety light and a room temperature ranging between 75^0^—80^0^ F. Two control films were used to determine the time of the radiographic film in the developer solution. The developer and fixer solutions were new and the water to wash the films between solutions was constantly drained during the process. After the radiographs were processed, they were scanned and digitalized using a Hewlett Packard model hp Scanjet 5470c. All radiographic films were transferred to Corel Office 2000 (Corel Print Office 5^®^), coded, de-identified and then stored in a Microsoft PowerPoint file (Microsoft^®^ 2010) for their interpretation. This process not only allowed to maintain the quality of the imagen but also to obtain a standardized method for the interpretation of the radiographs including the use of the same computer screen, light intensity, contrast, and size of the image.

The values used to score caries free teeth and caries in the enamel and dentin were based on the diagnostic criteria used by Otis and Sherman [35]. The scores and criteria to determine healthy teeth, as well as caries in enamel and dentin for the three systems are depicted in Table 1.

After the occlusal caries detection examinations were performed, a copy of the oral examination findings, indicating the teeth that needed dental treatment was delivered to each classroom’s teacher. The teachers were asked to give a copy of the oral examination findings to the parents making sure that they were informed of the need for dental treatment of their child. At the time of this study, The Department of Community Dentistry at UPCH, School of Dentistry provided community dental service in the educational institution where the study was performed and all children that participated in the study were part of this community dental service.

### Data processing

The data of each occlusal zone obtained with ICDAS II was entered into a hard copy format (Fig. 1a). For SCDS, the data was obtained from each zone of the occlusal surface’s image using a transparent template created for each type of tooth and superimposed on the computer screen so that the zones of the occlusal surface examined were consistently the same (Fig. 1b). Because the bitewing radiographs have two dimensions, and do not allow for direct caries detection on the occlusal surface, the data obtained from zone 2 (Fig. 1c) which includes the central, buccal, and lingual zones were compared with the values obtained from the central, buccal, and lingual zones of ICDAS II and SCDS. This modification reduced the bias and error in the comparative analysis among zones. The mesial and distal occlusal zones from the radiographic images were compared with the values obtained from mesial and distal occlusal zones of ICDAS II and SCDS. The values from the interproximal surfaces were not considered in the statistical analysis. To avoid bias and duplication of the information, the data entering process was performed independently for each method over several consecutive days. In all cases, the value entered for each zone on the occlusal surface was always the highest one.

The sensitivity and specificity of ICDAS II and RM were determined using SCDS as the reference test. The decision to have SCDS as the reference test was based on the high accuracy and reliability of LFS previously reported [15, 24, 36–39]. The sensitivity and specificity of ICDAS II were determined based on the detection of dental caries in the five zones of the occlusal surface. For RM, the sensitivity and specificity were determined using the modified format explained above.

### Statistical analysis

The data obtained from ICDAS II, SCDS and RM was transferred to an electronic version similar to the original hard copy format which was created using the EpiInfo program (CDC-EpiInfo™ 7). The data stored in the EpiInfo program was transferred to Excel 2010 (Microsoft^®^ Inc. 2010) and then to SAS 9.4 program for statistical analysis. The initial caries detection information obtained with the three methods was recategorized considering the criteria shown in Table 1. All identifiers were deleted, and a numerical code was assigned to each participant. Descriptive statistical analysis included the mean, standard deviation, and confidence interval at 95%. The inferential analysis to determine differences in the sensitivity and specificity between ICDAS II and RM was performed using the paired sample t-test for repeated measures with a significance level set at 5%.

## Results

Out of one hundred sixty-one participants, 160 children, 77 males and 83 females with a mean age of 9.42 and 9.45 years, respectively, completed all three caries detection exams. Six hundred thirty-two permanent first molars (maxilla: 315, mandible: 317), 364 primary first molars (maxilla: 179, mandible: 185), and 495 primary second molars (maxilla: 258, mandible: 237) were examined. Taking into consideration the three systems used in this study, a total of 22,365 occlusal zones were included in the analysis. Descriptive statistical analysis showed that the sensitivity and specificity values of ICDAS II and RM vary depending on whether the analysis is performed considering: (1) permanent and primary molars together, (2) primary molars together, (3) permanent or primary molars independently, and (4) whether the carious lesion is compromising the enamel or dentin (Table 2).

**Table 2:**
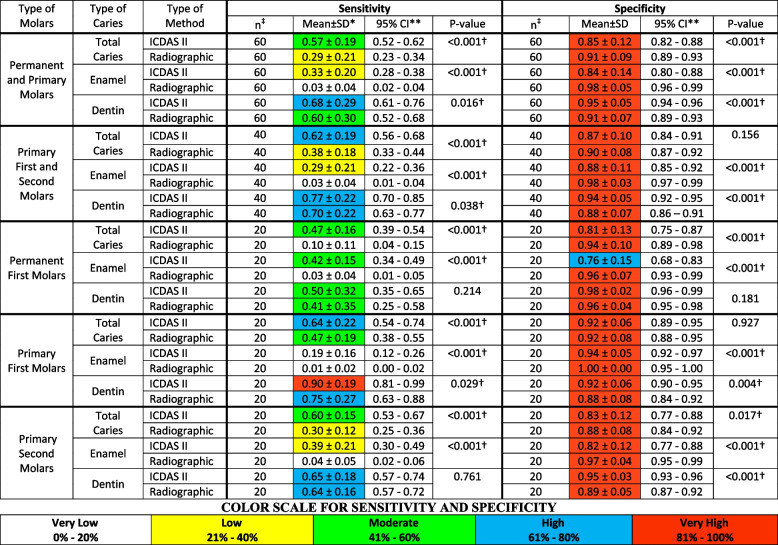
Comparison of the sensitivity and specificity values according to the type of tooth and caries detection method

^*^SD Standard deviation, ^**^CI Confidence interval

† paired sample t-test for repeated measures (*P* < 0.05)

‡ Number of occlusal zones to estimate sensitivity and specificity according to the type and number of teeth

The sensitivity and specificity were analyzed for all primary and permanent first molars together, and independently. For each category, the mean of the sensitivity and specificity of each occlusal zone was calculated and then averaged for total caries (caries in enamel and/or in dentin) and whether the caries were in enamel only or in dentin.

### Sensitivity

For total caries the mean and standard deviation of sensitivity with ICDAS II for all molars was 0.57 ± 0.19. The higher mean value was found in the primary first molars (0.64 ± 0.22) when compared to the permanent first molars (0.47 ± 0.16), and primary second molar (0.60 ± 0.15). When the carious lesions were stratified into caries in enamel only or in dentin the mean values were different. For caries in the enamel only, the mean for all molars was 0.33 ± 0.20. The permanent first molars showed the highest mean value (0.42 ± 0.15) in comparison to the primary molars together (0.29 ± 0.21), primary first molars (0.19 ± 0.16), and primary second molars (0.39 ± 0.21). The mean sensitivity for the primary and permanent first molars together with caries in dentin was 0.68 ± 0.29, with the primary first molars showing the highest value (0.90 ± 0.19) in comparison with all primary molars together (0.77 ± 0.22), the primary second molars (0.65 ± 0.18), and the permanent first molars (0.50 ± 0.32) (Table 2).

Overall, the sensitivity values with RM for total caries and caries in enamel were lower than those found with ICDAS II, however, the sensitivity of RM for caries in dentin for permanent and primary molars together (0.60 ± 0.30), primary molars together (0.70 ± 0.22), permanent molars only (0.41 ± 0.35), primary first molars (0.75 ± 0.27), and primary second molars (0.64 ± 0.16), showed a high to moderate sensitivity according to the color scale used in this study (Table 2). Except for caries in dentin for permanent first molars (*p* = 0.214), and primary second molars (*p* = 0.761), the sensitivity of ICDAS II for all categories was significantly higher than the one of RM (*p* ≤ 0.05).

### Specificity

When analyzing the specificity with ICDAS II, it was found that all values were very high. The specificity value for total caries was 0.85 ± 0.12. When the specificity was calculated for the primary and permanent first molars independently, the specificity was 0.87 ± 0.10 and 0.81 ± 0.13, respectively. The mean values of the specificity were also high when the carious lesions were stratified into caries in enamel only and those in dentin. The mean value for caries in the enamel for all molars was 0.84 ± 0.14. The mean value of the specificity for all primary molars together was (0.88 ± 0.11). Interestingly, when the specificity was estimated for the primary first and second molars independently, the mean values changed. Therefore, the first primary molars had the highest mean values (0.94 ± 0.05) when compared to primary second molars (0.82 ± 0.12) and permanent first molars (0.76 ± 0.15). The specificity values for caries in dentin were 0.95 ± 0.05 for all molars, 0.94 ± 0.05 for all primary molars together, 0.92 ± 0.06 and 0.95 ± 0.03 for primary first molars and second molars, respectively, and 0.98 ± 0.02 for permanent first molars. With RM, the mean value of the specificity for all categories was between 0.88 ± 0.07 and 1.00 ± 0.00 which is considered very high. The values of the sensitivity and specificity for the primary and permanent first molars, according to whether the carious lesion was compromising the enamel or dentin are shown in Table 2. The Specificity of RM was higher for the total caries when all molars were combined, and for permanent first molars only (*p* < 0.001). Although the mean specificity of RM was higher for the total caries in the primary molars together, there was no statistical difference when compared to ICDAS II (*p* = 0.156), however, the mean was higher for primary second molars alone (*p* = 0.017). The specificity of RM for caries in enamel was higher than ICDAS II in all categories (*p* < 0.001). The specificity of ICDAS II for caries in dentin was significantly higher when all molars were combined (*p* < 0.001), for all primary molars together (*p* < 0.001), and for primary first molars (*p* = 0.004), and second primary molars (*p* < 0.001). Even though the mean specificity for caries in dentin of ICDAS II was higher in the permanent molars, there was no statistical difference when compared to RM (*p* = 0.181).

## Discussion

To our knowledge, this is the first in vivo study designed to compare the sensitivity and specificity of ICDAS II and RM in detecting dental caries based on five zones of the occlusal surface of primary first and second molars, and permanent first molars having SCDS as the reference test. The decision to use the SCDS as the reference test was based on the results of in-vitro studies which have demonstrated that the performance of SCDS is similar to the clinical and radiographic methods with a receiver operating characteristic curve of 0.82 [26, 40]. Most recently, these findings have been supported by the diagnostic accuracy in detecting incipient enamel and dentin occlusal carious lesions [27].

In the present study, the results of the sensitivity and specificity values showed that the RM and ICDAS II varied according to both the type of carious lesion and tooth examined. An important component of this study was to perform an accurate interpretation of the radiographic images. For this reason, all bitewing radiographs taken using a conventional method were scanned and digitalized. Although this step helped avoid errors and be more precise in the radiographic interpretation, the results of this study would not be different if a conventional radiographic method was used instead [41]. Thus, RM showed low sensitivity, but a high specificity in teeth with carious lesions that were compromising the enamel. An explanation for this would be the superimposition of the image obtained from the bitewing radiographs. If a carious lesion is affecting the central-occlusal zone, it is possible that due to the size and depth of the carious lesion it can be hidden by healthy tooth tissue from the occlusal-lingual and occlusal-buccal zones and be interpreted as a caries-free zone. The low values of sensitivity and high values of specificity of RM found in this study are similar to those reported by in vivo and in vitro studies [20, 26, 42].

It has been noted in previous studies that the sensitivity of RM to detect caries in the enamel is quite low, but very good for detecting caries in dentin and caries in the inter-proximal surfaces [43, 44]. These findings suggest that bitewing radiographs are not the best system to detect incipient occlusal caries and should not be used as an indicator to establish preventive treatment. For instance, the prevalence of carious lesions in enamel may be underestimated and not considered as part of the problem.

The sensitivity of ICDAS II for all teeth examined in this study showed values lower than those reported by in vitro and in vivo studies [20, 37, 39, 42, 45]. The discrepancy may be because in this study, all comparisons were made in the same occlusal zone examined, which differs from other studies that do not indicate with certainty whether the comparison was from the same area where the carious lesion was detected, suggesting that the results of those studies could have had a bias related to the location of the examined zone, and therefore may not be accurate. This study not only demonstrated that the sensitivity and specificity values vary according to the type of tooth examined, but also whether the carious lesions were interpreted as a total caries (enamel and dentin together) or independently as caries in enamel only or in dentin.

According to the color scale, the mean values of specificity for the enamel and dentin were very high for ICDAS II and RM. In contrast, the sensitivity of ICDAS II and RM showed different mean values for total caries, as well as for caries that compromise the enamel or dentin of primary first and second molars, and permanent first molars. These differences suggest that the sensitivity must be estimated based on the type of tooth and whether the detection of caries is compromising the enamel or dentin. The differences might also be related to the age of the children included in the study, and the fact that primary molars had a higher prevalence of caries, particularly in cases of caries that compromise the dentin. On the latter point, it has been suggested that the prevalence of the disease may have a direct influence on the sensitivity and specificity values of a diagnostic test [46]. The prevalence of caries found in this study varied depending on the system used. Therefore, using the ICDASII, RM, and SCDS, the prevalence of caries was 96.9%, 92.5% and 100%, respectively. Whether these differences have influenced the results found in this study is dependent on an analysis that was out of the scope of this study.

Another interpretation of these differences may be associated with (1) the irregular anatomy of the occlusal surface making the visual examination more difficult, (2) the presence of non-cleansable deep occlusal pits and fissures, (3) the estimation of the sensitivity which was based on combining all primary molars and first permanent molars, and independently for each type of molars, (4) the highest capacity of the SCDS to detect caries in enamel and dentin, (5) the estimation of the sensitivity in vivo and in real time which differ substantially from in vitro studies, and (6) the calibration of the examiner to detect occlusal caries.

This study shows the direct results of the sensitivity and specificity in vivo, which are based on the natural and biological status of occlusal caries in children. Compared with other in vitro studies, this study controlled the confounders associated with the methods used to store and prepare teeth which have been shown to change the optical properties of the tooth, the concentration of fluorophores, and the fluorescence values of LFS [47, 48].

Although the determination of the sensitivity and specificity of a test or system are important epidemiological indicators to differentiate between the true positive and true negative cases, it may also allow for the establishment of preventive programs and prioritizing resources specifically oriented for people from underserved communities who need preventive treatment, thus avoiding reparative treatments which are invasive and associated with an increase in the cost of healthcare services.

This study has shown that sensitivity and specificity of the ICDAS-II and RM for detecting occlusal caries having the SCDS as the reference test varies according to the type of molars examined and whether the caries lesion is compromising the enamel or dentin.

## Conclusions

Using the SCDS as the gold standard, the values of sensitivity of the ICDAS II and RM for detecting caries in dentin ranged from moderate to very high, whereas their sensitivity for detecting caries in enamel was very low to low. Regarding the specificity, both methods show values considered high and very high for detecting caries in the enamel or in dentin of the primary first and second molars, and permanent first molars. This finding may be related to the subjectivity in detecting caries when the ICDAS II and RM are used, and to the optical properties of the SCDS system which gives an objective result in the detection of caries. In any case, both the subjective and objective components of the method used, which are inherent in any study must be controlled throughout a good calibration process.

### Recommendations

The values of sensitivity and specificity of the ICDAS II and RM in detecting occlusal caries should be estimated according to the type of molars examined, and whether the carious lesion is compromising the enamel or dentin of the primary first and second molars or first permanent molars.

## Data Availability

The data generated in the study is not publicly available because there are other variables in the study that are still under analysis and interpretation but are available from the corresponding author on reasonable request.

## Abbreviations

ICDAS II: International caries detection and assessment system
LFS: Light fluorescence system
RM: Radiographic method
SCDS: Spectra™ caries detection system
UPCH: Universidad Peruana Cayetano Heredia
VE: Visual examination method

## References

[1] Gomez J, Zakian C, Salsone S, Pinto SCS, Taylor A, Pretty IA, Ellwood R (2013). In vitro performance of different methods in detecting occlusal caries lesions. J Dent.

[2] Ferreira Zandoná A, Zero DT (2006). Diagnostic tools for early caries detection. JADA.

[3] Parviainen H, Vähänikkilä H, Laitala ML, Tjäderhane L, Anttonen V (2013). Evaluating performance of dental caries detection methods among third-year dental students. BMC Oral Health.

[4] Ismail AI, Sohn W, Tellez M, Amaya A, Sen A, Hasson H, Pitts NB (2007). The International Caries Detection and Assessment System (ICDAS): an integrated system for measuring dental caries. Community Dent Oral Epidemiol.

[5] Sebastian ST, Johnson T (2015). International Caries Detection and Assessment System (ICDAS): An Integrated Approach. Int J Oral Health Med Res.

[6] Jablonski-Momeni A, Stachniss V, Ricketts DN, Heinzel-Gutenbrunner M, Pieper K (2008). Reproducibility and Accuracy of the ICDAS-II for Detection of Occlusal Caries in vitro. Caries Res.

[7] Braga MM, Oliveira LB, Bonini GAVC, Bönecker M, Mendes FM (2009). Feasibility of the International Caries Detection and Assessment System (ICDAS-II) in Epidemiological Surveys and Comparability with Standard World Health Organization Criteria. Caries Res.

[8] Jablonski-Momeni A, Ricketts DNJ, Heinzel-Gutenbrunner M, Stoll R, Stachniss V, Pieper K (2009). Impact of Scoring Single or Multiple Occlusal Lesions on Estimates of Diagnostic Accuracy of the Visual ICDAS-II System. Int J Dent.

[9] Haak R, Wicht MJ, Noack MJ (2001). Conventional, digital and contrast-enhanced bitewing radiographs in the decision to restore approximal carious lesions. Caries Res.

[10] Gowda S, Thomson WM, Foster Page LA, Croucher (2009). What difference does using bitewing radiographs make to epidemiological estimates of dental caries prevalence and severity in a young adolescent population with high caries experience?. Caries Res.

[11] Hopcraft MS, Morgan MV (2005). Comparison of radiographic and clinical diagnosis of approximal and occlusal dental caries in a young adult population. Community Dent Oral Epidemiol.

[12] Harper R (2002). Can sensitivity and specificity estimates from research studies be made more meaningful for clinical practice?. Ophthal Physiol Opt.

[13] Trevethan R (2017). Sensitivity, Specificity, and Predictive Values: Foundations, Pliabilities, and Pitfalls in Research and Practice. Front Public Health.

[14] The Swedish Council on Technology Assessment in Health Care. “Karies diagnostik, riskbedomning och icke-invasiv ¨ behandling”. 2007;188:84.

[15] Gimenez T, Braga MM, Raggio DP, Deery C, Ricketts DN, Mendes FM (2013). Fluorescence-Based Methods for Detecting Caries Lesions: Systematic Review, Meta-Analysis and Sources of Heterogeneity. PLoS ONE.

[16] Yu OY, Zhao IS, Mei ML, Lo E, Chu CH (2017). A Review of the Common Models Used in Mechanistic Studies on Demineralization-Remineralization for Cariology Research. Dent J.

[17] Özkan G, Kanli A, Başeren NM, Arslan U, Tatarİ. Validation of micro-computed tomography for occlusal caries detection: an in vitro study. Braz Oral Res. 2015;29(1):S1806- 83242015000100309.10.1590/1807-3107BOR-2015.vol29.0132. 10.1590/1807-3107BOR-2015.vol29.0132 26892360

[18] Pretty IA (2006). Caries detection and diagnosis: Novel technologies. J Dent.

[19] 22. Rechmann P, Charland D, Rechmann BMT, Featherstone JDB. Performance of laser fluorescent devices and visual examination for the detection of occlusal caries in permanent molars. J Biomed Opt, March 2012;17(3). Available at: https://www.spiedigitallibrary.org/journals/Journal-of-Biomedical-Optics”. Accessed: 2021- 10.1117/1.JBO.17.3.036006 22502564

[20] Diniz MB, Boldieri T, Rodrigues JA, Santos-Pinto L, Lussi A, Cordeiro RC (2012). The performance of conventional and fluorescence-based methods for occlusal caries detection: an in vivo study with histologic validation. J Am Dent Assoc.

[21] Seremidi K, Lagouvardos P, Kavvadia K (2012). Comparative in vitro validation of VistaProof and DIAGNOdent pen for occlusal caries detection in permanent teeth. Oper Dent.

[22] Souza JF, Boldieri T, Diniz MB, Rodrigues JA, Lussi A, Cordeiro RC (2013). Traditional and novel methods for occlusal caries detection: performance on primary teeth. Lasers Med Sci.

[23] Melo M, Pascual A, Camps I, Del Campo A (2015). In vivo study of different methods for diagnosing pit and fissure caries. J Clin Exp Dent.

[24] Kockanat A, Unal M (2017). In vivo and in vitro comparison of ICDAS II, DIAGNOdent pen, CarieScan PRO and SoproLife camera for occlusal caries detection in primary molar teeth. Eur J Paediatr Dent.

[25] Muller-Bolla M, Joseph C, Pisapia M, Tramini P, Velly AM, Tassery H (2017). Peformance of a recent light fluorescent device for detection of occlusal caries lesions in children and adolescents. Eur Arch Paediatr Dent.

[26] Graye M, Markowitz K, Strickland M, Guzy G, Burke M, Houpt M (2012). In vitro evaluation of the Spectra early caries detection system. J Clin Dent.

[27] Salama M, Hassanein O, Shaalan O, Yassen A (2022). Clinical effectiveness of high definition fluorescence camera in detection of initial occlusal caries. J Clin Exp Dent.

[28] De Benedetto MS, Morais CC, Novaes TF, Rodriguez JA, Braga MM, Mendes FM (2011). Comparing the reliability of a new fluorescent camera with conventional laser fluorescent devices in detecting caries lesions in occlusal and smooth surfaces of primary teeth. Laser Med Sci.

[29] Chapman J, Bachand D, Hyrka SK (2011). Testing the sensitivity, specificity and feasibility of four falls risk assessment tools in a clinical setting. J Nurs Manag.

[30] Baelum V, Heidmann J, Nyvad B (2006). Dental caries paradigms in diagnosis and diagnostic research. Eur J Oral Sci.

[31] Gomez J (2015). Detection and diagnosis of the early caries lesion. BMC Oral Health.

[32] Instituto Nacional de Estadística e Informática. Encuesta demografica y salud familiar- 2021.Series anuales de indicadores principals de la ENDES, 1986–2021. Publication available at: https://www.inei.gob.pe/media/MenuRecursivo/publicaciones_digitales/Est/Lib1841/libro.pdf. Accessed:2022–05–22.

[33] Edelman DJ (2018). Managing the Urban Environment of Lima, Peru. Advances in Applied Sociology.

[34] International Caries Detection and Assessment. Available at: “http://www.icdas.org/elearning- program”. Accessed: April 12, 2016.

[35] Otis L, Sherman R (2005). Assessing the accuracy of caries diagnosis via radiograph-film versus print. J Am Dent Assoc.

[36] Jablonski-Momeni A, Liebegall F, Stoll R, Heinzel-Gutenbrunner M, Pieper K (2013). Performance of a new fluorescence camera for detection of occlusal caries in vitro. Lasers Med Sci.

[37] Jablonski-Momeni A, Stucke J, Steinberg T, Heinzel-Gutenbrunner M. Use of ICDAS-II, Fluorescence-Based Methods, and Radiography in Detection and Treatment Decision of Occlusal Caries Lesions: An In Vitro Study. Int J Dent 2012; 8 pages Article ID 371595. 10.1155/2012/371595. 10.1155/2012/371595 PMC3437738 22973311

[38] Javed F, Romanos GE (2015). A comprehensive review of various laser-based systems used in early detection of dental caries. Stoma Edu J.

[39] Rodrigues JA, Hug I, Diniz MB, Lussi A (2008). Performance of fluorescence methods, radiographic examination and ICDAS II on occlusal surfaces in vitro. Caries Res.

[40] Markowitz K, Gutta A, Merdad H, Guzy G, Rosivack G (2005). In Vitro Study of the Diagnostic Performance of the Spectra™ Caries Detection Aid. J Clin Dent.

[41] Abesi F, Mirshekar A, Moudi E, Seyedmajidi M, Haghanifar S, Haghighat N, Bijani A (2012). Diagnostic accuracy of digital and conventional radiography in the detection of non-cavitated approximal dental caries. Iran J Radiol.

[42] Diniz MB, Lima LM, Eckert G, Zandona AG, Cordeiro RC, Santos Pinto LS (2011). In vitro evaluation of ICDAS and radiographic examination of occlusal surfaces and their association with treatment decisions. Oper Dent.

[43] Wenzel A (2004). Bitewing and digital bitewing radiography for detection of caries lesions. J Dent Res.

[44] Schwendicke F, Tzschoppe M, Paris S (2015). Radiographic caries detection: A systematic review and meta-analysis. J Dent.

[45] Kouchaji C (2012). Comparison between a laser fluorescent device and visual examination in the detection of occlusal caries in children. Saudi Dent J.

[46] Leeflang MM, Rutjes AW, Reitsma JB, Hooft L, Bossuyt PM (2013). Variation of a test’s sensitivity and specificity with disease prevalence. CMAJ.

[47] Francescut P, Zimmerli B, Lussi A (2006). Influence of different storage methods on laser fluorescence values: A two-year study. Caries Res.

[48] Burin C, Burin C, Loguercio AD, Grande RHM (2005). Occlusal caries detection: a comparison of a laser fluorescence system and conventional methods. Pediatr Dent.

